# On the suitability of phillipsite-chabazite zeolitite rock for ammonia uptake in water: a case study from the Pescara River (Italy)

**DOI:** 10.1038/s41598-022-13367-y

**Published:** 2022-06-03

**Authors:** Daniela Novembre, Domingo Gimeno, Monia Calista, Vania Mancinelli, Enrico Miccadei

**Affiliations:** 1 Dipartimento di Ingegneria e Geologia, Università degli Studi “G.D’Annunzio”, Via dei Vestini 30, 66013 Chieti, Italy; 2 Department of Mineralogia, Petrologia i Geologia Aplicada, Universitat de Barcelona, 08028 Barcelona, Spain

**Keywords:** Environmental sciences, Planetary science

## Abstract

Ionic exchange tests have been performed on superficial wastewaters to remove ammonia using a volcanic zeolitized rock from Lazio Region (Central Italy). The zeolitite (natural zeolite) is characterized by chabazite, phillipsite and minor amounts of sanidine, leucite and analcime. After preliminary column experiments in laboratory focused to determine the saturation time of the zeolitite, a pilot plant was built up on a little water course near the area of San Giustino channel (Abruzzo Region, Central Italy). Wastewaters, characterized by starting ammonia value ranging between 5 and 120 mg/l, were filtered with a zeolitic bed. The first experimental results indicate a positive ammonia reduction of about 80–90% and, in all cases, NH^4+^ concentration values under the EU law limits. A main purpose of this paper is to evidence that most of studies published on uptake of ammonia by means of zeolitite lead with clinoptilolite-dominant zeolitite despite the large and best performance of phillipsite-chabazite zeolites (up to 61–79% improvement of ammonia uptake). Last but not least, a large number of published studies are of difficult comparison because of poor characterization of the zeolitite used.

## Introduction

Nitrogen is a requisite and a highly demanded element for living organisms on Earth. Despite this, geogenic ammonia in superficial and groundwaters are usually below 0.2 mg/L^1^, even if in the Mediterranean area contents reaching up to 0.6–1 mg/l are retained as good to fair indicators of water quality^2^. However, increase in human activities have greatly altered the global nitrogen cycle, especially in rivers and streams, resulting in eutrophication, formation of hypoxic zones, and increased production of NO_2_^3^.

Ammonia reduction from superficial wastewaters is an environmental problem related to a number of anthropic activities, including among others residual urban waters (sanitary, grey waters, etc.), intensive cattle raising, and fish aquaculture. Natural zeolite has been largely tested and used as a cheap and accessible product for ammonia uptake, and eventually associated to N and P precipitation and valorization as fertilizer.

Ammonium ion concentrations are at least one order of magnitude higher in municipal wastewaters than in natural waters and inorganic and organic impurities also occurs in greater amounts^4^. A large number of countries have developed environmental legislation along the last decades. In the European Union this has been unified by the Council Directive 91/676/CEE^5^, that has been successively implemented by the single countries. Just to show the case of Italy (were the case study here exposed occurs) a good environmental protection was stablished by the so-called “Legge Merli”, where the the limit value for NH4^+^ concentration in sewage waters is 15 mg/l^6^. Higher concentration values might be detected in natural superficial waters, mainly due to the agricultural use of the land^7^. Then, this law was enlarged and improved by several laws and govern decrets, till the European Union directive^5^ has been finally incorporated and detailed^8^. The result is an extremely detailed explanation of each case (also specifying facts like the analytical procedure to be underwent for each analyte studied) but finally the legal limit for emission for ammonia in superficial waters remain the same (15 mg/L) while for sewerage are twice (30 mg/L). WHO^9^ also agrees with the 15 mg/L threshold. Furthermore, we can note that European legislation is very detailed with reference to the requisites of the portfolio declaration of environmental impact in anthropized areas.

It is out of the scope of this paper to compile a dataset of the legal limits everywhere; we can simply remark that similar legal thresholds can be found in a large number of countries, and that from case to case more emphasis focus on un-ionized or ionized ammonia. Also, some national environmental agencies and ministries remark toxicological effects and relative threshold on biota^10,11^. Thus, we can conclude that we take the European legal thresholds for ammonia as a reference value in this paper, and ammonia surplus due to anthropic activities is widespread in a large number of natural environments and his uptake and mitigation become a matter of great interest everywhere.

In the last decades experimental studies for ammonia nitrogen removal from water increasingly improved. Ammonium compounds are characterised by an extreme solubility in the presence of water; for this reason, their removal requires complex treatments: biologic treatments (nitrification and denitrification, biodegradation), chemical treatments (oxidation, chlorination), electrochemical^12^ and chemical-physical treatments (ionic exchange)^13–16^. The biological process is commonly considered a cost-effective and efficient treatment process for domestic wastewater^17^. However, nitrification and denitrification activities are inhibited at low temperature (i.e., under 14 °C), making biological processes unfit to meet the strict disposal limits in cold regions^18^.

The most common option for ammonia removal is the break point chlorination^19^. Even if effective, water treatment with this method drives to the formation of organic-halogen compounds characterized by carcinogenic effects.

As far as chemical-physical treatments are concerned, zeolites are nowadays referred as ideal materials^20–22^. Zeolites are hydrates aluminosilicates of alkaline and alkaline-earth metals, whose structure is essentially made of a three-dimensional framework of SiO_4_ and AlO_4_ tetrahedrons. The Al^3+^ substitution with Si^4+^ causes a negative charge in the lattice usually balanced by extra framework cations, the so-called exchangeable cations (Na, K, Ca, etc.), that can be easily replaced by other cations^23^. Regarding this, application of zeolites in water purification processes is based on the high affinity of these minerals respect to cations^24^, which are substituted into their structure by means of ionic exchange processes. This property, known as selectivity in ionic exchange, is well testified for clinoptilolite, phillipsite, chabazite and other zeolites, showing these minerals a high affinity for the NH^4+^ ion^4,16,25,26^. For this reason (and economic ones), zeolites are nowadays preferred above synthetic resins, for which ionic exchange is instead not a selective property.

In most of cases reported in technical literature, ammonia uptake by natural zeolite is referred to clinoptilolite-mordenite zeolitites. For this reason, we test here the efficacy for ammonia uptake of an italian chabazite-phillipsite zeolitite the so-called “Tufo Rosso a Scorie Nere” (TRS)^27,28^, coming from the Latial Province (Central Italy), used in the regeneration of sewage waters of the San Giustino channel, a tributary of the Pescara River (Abruzzo Region, Central Italy). Preliminary experiments based on continuous column process were performed in laboratory. Furthermore, a main purpose of the paper is to focus attention of the potential use of the widespread chabazite-phillipsite zeolitite resources in ammonia removal from sewage waters everywhere.

### Some geological facts: zeolitite and zeolite, rock and minerals, this is not the same

Most people involved in water treatment have not a geological formation, since this field of research is mostly related to engineering chemists, chemists, biologists, etc. This explains some basic concepts commonly misunderstood arising in the analysis of available bibliography on this subject. These concepts are of capital importance in the comprehension of the suitability of each specific zeolitic product in the removal as cation exchanger of ammonia (and a number of other products); there are basic how know for a geologist, but evidence shows that they are not for all professionals concerned with water depuration.

Zeolites constitute a group of minerals (silicates corresponding to the category of tectosilicates) that is characterized by a tridimensional structure based on the assembly of silicon tetrahedra (that is an atom of silica at the center of a tetrahedron with four oxygen atoms at the vertex) that share several oxygen atoms with other tetrahedra, allowing the construction of the tridimensional structure of these minerals. The exchange capacity of zeolite minerals is related to the fact that some silicon atoms are substituted by aluminum ones, a fact that provokes electrochemical instability that is balanced with cations. The tridimensional structure of zeolites related to other tectosilicates (as quartz or feldspar) is expanded, with large channels and wide inner cavities, of size specific of each zeolite minerals, a fact that also allows using zeolites as molecular sieves.

The first thing that needs to be pointed out is the difference between the term zeolite and zeolitite. Zeolites were first synthetized in the late 40’s of the XX century^29^, obtaining industrial monophasic products, and this fact as allowed for a great developing of a great number of applications. On other hand, natural monophasic zeolites are the exception, and bi- or polymineralic rocks are the rule in ore deposits. Considering the microcrystalline character of these rocks, and the very similar physical properties of the different zeolite minerals, nobody can envisage an economic preparation procedure in order to obtain monomineralic samples for natural bi- or polymineralic rocks. Thus, the first consequence of this is when you read in a technical or scientific paper or in the leaflet “natural zeolite” (the mineral) of a supplier, you might simply read “zeolitite” (the rock mainly made with natural zeolite minerals).

Most of commercial brochures (but also technical papers) offer as main (or only) characteristics of a zeolitite (“natural zeolite”) its chemical composition. This might be good (or at least enough) for a synthetic monomineralic zeolite. Also, in a general way, chemical characterization is a good approach in most of industrial rocks. In example, chemical composition of a carbonate or a feldspar rock is a good proxy for its mineralogical features and industrial applications, since there are little mineralogical changes related to chemical differences and/or the industrial process concerned destroys the crystallochemical lattices of minerals and provides reactants that can be considered in its oxide equivalent. Thus, a carbonate rock can contain a wide percentual variation of several mineral species (calcite, aragonite, dolomite, ankerite, siderite, etc.). But these differences are not chemically relevant (i.e. calcite and aragonite are polymorphs of calcium carbonate) or otherwise they can easily be modelled taking into account a few chemical oxides (i.e., MgO can be assigned to dolomite, calcium-magnesium carbonate, of fixed stoichiometric composition, since magnesite—magnesium carbonate—is very rare in nature). Also, the industrial uses of carbonates commonly lead to the destruction of the carbonate phases (i.e. by calcination in cement industry, or by dissolution in remineralization in desalination processes of water^30^). In the same way, feldspar rock or mineral feldspar concentrates can be modelled by the oxide content of alkalis (Na_2_O and K_2_O) since its end users (glass industry, ceramic industry, etc.) are mainly concerned with the flux behaviour of a mainly silica-constituted mix at high temperature in the kiln (and this depends on the total alkali oxides content).

Otherwise, most zeolitite rocks are made of minerals that are aluminosilicates with variable contents of alkaline and alkaline-earth elements (Na, K, Ca, Mg, etc.). These minerals are very difficult to be easily transferred to percentage mineral contents from chemical oxide data. In this sense, a rough table of chemical data of zeolitites simply offers in the best of cases a general idea of the original rock from the zeolitite is derived (see below on the genesis of zeolitite deposits). In the specific case of zeolitites used for cation exchange, in the best of cases the supplier provides the cation exchange capacity (CEC) based on theoretical formulae^31^ (a fact that is usually misleading) or in empirical tests^32^. This value is just a generic indication, since the main characteristic of the zeolite group of minerals is the specificity of each specie with reference to the cation exchange, a fact that is enhanced by a large number of specific studies of cation uptake comparing zeolites from a large number of occurrences or suppliers. In practical terms, it is very difficult to compare in a precise way the behaviour of zeolitites studies in different papers (and this justifies pro-parte the use of empirical CEC as an indication of zeolitite performance).

Even though it is possible the genesis of natural zeolites in a large variety of geological environments^33^, minable-sized deposits are mainly related to two situations: the transformation of glass-rich volcanoclastic deposits and the genesis of chemical sediments within alkaline lakes (and in this case, the source of silica and aluminum frequently is volcanic glass again). Experimental work confirms that the temperature of formation is lesser than 200 ºC and the glass reaction or dissolution occurs under low-pressure conditions. Volcanic glass is metastable, and the growth of zeolite minerals at the expenses of glass can be a syngenetic process related to the emplacement and early cooling of pyroclastic (explosive volcanism) deposits (the so-called geoautoclave phenomena^34^) or a late or ever very late diagenetic phenomenon related to the infiltration of meteoric of hydrothermal waters. In any case, a complete cross-section of a minable deposit commonly shows lateral and vertical variation of the mineral paragenesis, that contains a continuous reaction of neo formed zeolite minerals (and sometimes, clays) as well as the relictic unaltered silicates (quartz, feldspars, and eventually other silicates) of the volcanic glass fragments or coming from the detritic fraction of the sediment^35^.

The Si/Al relationship in the starting material, and the pH of the involved water solutions largely control the zeolite mineral formed^36^. Thus, in the case of zeolitites generated starting from silica-rich volcanic glass (in petrology, the so-called silica oversaturated glass, meaning that alkaline and alkaline-earth are not enough to consummate all the available silica in feldspar formation in the case of complete magmatic crystallization) the zeolitites formed are mostly clinoptilolite-mordenite rich. In the opposite side relatively silica-poor glass (the so-called silica subsaturate glass, meaning that in the case of complete magmatic crystallization the silica is not enough to capture all available alkaline and alkaline earth elements in feldspar crystalline lattices) lead mostly to form phillipsite and chabazite-rich zeolitites. Other neo formed zeolite minerals, like analcime, gismondine, natrolite, etc. commonly occurs like minor components of zeolitite.

In practical terms, a zeolitite exploitation should tend to mostly mine rocks formed by two main mineral phases, and in the ideal case with one mineral phase largely dominant over the other. If the mineral zonation of the deposit occurs as stratiform-like bodies of rock (in pyroclastic volcanic rocks) or true sedimentary strata characterized by a dominant phase, producers might be fully conscientious of this fact, and mine separately each different zeolitite rock. Also, processing of each type of minable ore should be done taking care of reiterate mixing and homogenization of each individual type of ore, in order to warrant an uniform final product for each type of zeolitite. Otherwise, if morphology or the zeolitite deposits (and their macroscopic features) do not allow for obtaining separate zeolitite products, special care of homogenization of the only one product obtained in an open pit should be conducted. Taking into account that just in this way a raw material supplier can warrant a compositionally stable source of zeolitite, then each producer should be develop periodic quantitative XRD characterization in order to maintain a homogeneous source of zeolitite to the end users. Such a procedure might allow i.e. for the approximate estimation of the real CEC of each commercial product, since theoretical CEC are just referred to pure monomineralic phases.

Bibliographic analysis of papers related with ammonia uptake form water (see Appendix A) show that in most of cases the zeolitite involved in these processes are clinoptilolite rich, or in lesser degree mordenite rich polimineralic rocks. This is striking, since from some decade ago the suitability of phillipsite and chabazite for ammonia uptake and removal from water is known in mainstream publications^37–39^. This suitability is not rare, if we consider the pore size of these zeolites and the molecular dimension of ammonia^40,41^. In Italy, the zeolitite rocks correspond to many pyroclastic volcanic units mainly related to the potassic alkaline province that are mined for building purposes at great scale since Etruscan and Roman times till today (see in example^42–44^ and references therein). Italian zeolite suppliers recycle sawing waste of dimension stone blocks for the quarries; and Italian researchers have prospected since 60’s of XX century industrial uses of these zeolitites. Most of the original volcanic rocks in these Italian districts were silica-undersaturated with respect to alkali and Ca present in the magmas, trachytic in composition. One could suspect that these rocks are rare in nature, since the Italian potassic alkaline province was considered along a century a rare magmatic association, but in fact trachytic pyroclastic rocks are frequent in several petrologic situations, and pyroclastic (and also subacqueous hydroclastic) basaltic rocks are also good potential sources of fragmental easily exploitable glassy rocks.

Therefore, it might be envisaged their use in several favourable geological contexts where zeolitite should be largely available. We can consider for instance: oceanic volcanic islands (like Hawaiian and Canary islands^45^), specially “old” mature volcanic islands where chemically evolved magmas erupt in form of large-sized pyroclastic rocks^47^; pristine arc-island environments or thin immature continental crustal segments (like large segments in the Panamá-Guatemala region in Centro America); in pyroclastic sectors of the large intraplate flood basalt regions^48^ or other within plate large basaltic outcrops^49,50^; and of course in the only case of emerged mid-ocean ridge, Iceland (where widespread hydrothermal metamorphism in zeolite facies is well exposed in the marginal, older, eroded sectors of the island^51^). Thus, we can conclude that there is not availability a reason for the margination of phillipsite-chabazite zeolitites from industrial uses.

Considering the four most frequent natural zeolites Colella^44^ demonstrated that selectivity for ammonia is comparable (but higher for the former) between chabazite and clinoptilolite, and much higher in the case of phillipsite, been the worst results in the case of mordenite, a fact that is also evident in CEC based on theoretical formulae^31^. In the specific case of water treatment for ammonia uptake and removal, phillipsite is considered better than clinoptilolite, a fact that even leaded to the obtention of patents of synthesis of phillipsite (see^37^ and references therein). Even, the fact that phillipsite is much more efficient for ammonia removal lead to obtain phillipsite by hydrothermal modification of a clinoptilolite-mordenite zeolitite^52^, obtaining a product that took up twice the amount of ammonium ions as the starting material. This might be not surprising since^53^ calculated the cation exchange capacity (CEC) of some common zeolites, based on their theoretical formulae, and showed that the ones of phillipsite (3.87 meq/g) and chabazite (3.70 meq/g) are much higher than the respective for mordenite (2.29 meq/g) and clinoptilolite (2.16 meq/g).

It has been calculated^54^ the Na^+^/NH^4+^ cation-exchange isotherms for phillipsite from Neapolitan yellow tuff from Italy demonstrating that phillipsite is more selective for NH^4+^ than clinoptilolite from Hector, California. Regarding this point, Italian pyroclastic rocks, like the “Tufo Giallo Napoletano” and “Tufo Rosso a Scorie Nere”, containing chabazite and phillipsite, have usually been tested for the ammonia removal from sewage urban waters, leather industry waters, zootechnical farming, aquaculture, and water purifying^7,54,55^. For example, it has been tested^56^ an Italian tuff containing chabazite and phillipsite in the treatment of wastewaters from swine sewage and found that the effective NH^4+^ exchange capacities of the zeolite rich material ranged from 0.4 to 0.9 meq NH^4+^/g. Other authors^46^ have proved that a glassy-rich zeolitite having a content as reduced as 20% of phillipsite results in good ammonia, phosphate and soluble organic matter retention in urban wastewater from Santa Cruz de Tenerife city (Canary Islands).

Some studies^57^ also demonstrated that phillipsite has more effectiveness in the case of ClNa-exchanged and after regeneration. This fact is also of great importance, since exhausted zeolites can be used as nitrogen fertilizers in agriculture, or ammonia exchanged NaCl solution can be purified obtaining MgNH_4_PO_4_ utilizable as a high premium quality slow-release solid fertilizer (^57^ and references therein). Some recent works emphasize the great significance of new economic routes for production of ammonium-based fertilizers from wastewaters^58^ using membranes and other physical barriers, as the enhanced recover of N and faster rate of nitrification when chabazite or other similar zeolites are involved in the process^59^. Nevertheless, these topics on regeneration of zeolitite and N recover as fertilizer is out of the scope of this paper.

## Materials and methods

Zeolitite used in this study is the “Tufo Rosso a Scorie Nere” (TRS), (^27,28^ and references therein) the largest pyroclastic flow related to the ancient volcano of Vico, a deposit that extends on a surface of about 1300 km^2^ This pyroclastic flow deposit is essentially massive and commonly made of black vitreous vesiculated juvenile elements immersed inside a yellow zeolitized ash matrix^27,28^. TRS rock was finely ground (particle size < 60 µm) and analysed by powder X-ray diffraction (PXRD) on a Siemens D5000 diffractometer operating with Bragg–Brentano geometry; CuKα = 1.518 Å, 40 kV, 40 mA, 2°–45° 2θ scanning interval, step size 0.020° 2θ) (Fig. C1, Appendix C). Both crystalline and amorphous contents of TRS were estimated using quantitative phase analysis (QPA) applying the combined Rietveld and reference intensity ratio (RIR) methods; corundum NIST 676a was added to each sample, amounting to 10%, according to the strategy proposed by^60^, and the powder mixtures were homogenized by hand-grinding in an agate mortar. Data for the QPA refinement were collected in the angular range 5–70 2theta (Fig. C2, Appendix C) with steps of 0.02° and 10 s step-1 (as previously developed^61,62^). Data were processed with GSAS software^63^ and its graphical interface EXPGUI^64^.

The cation exchange capacity (CEC) was determined using the ammonium acetate method^65^.

The composition of the zeolitite obtained from the average of 15 analyses (Table 1) was determined by X-ray fluorescence (XRF), with a Sequential X-Ray Spectrophotometer PHILIPS PW 2400. Major elements determination has been carried out using fused pearls (lithium tetra borate pearls at a dilution 1/20).

**Table 1:** Chemical composition of TRS.

		St. dev
SiO_2_	51.69	0.12
TiO_2_	0.45	0.01
Al_2_O_3_	18.15	0.09
Fe_2_O_3_	3.57	0.05
MnO	0.11	0.01
MgO	1.18	0.04
CaO	4.0	0.08
Na_2_O	1.85	0.03
K_2_O	5.75	0.08
P_2_O_5_	0.20	0.01
loi	12.57	0.11

The data shown are the result of the average of 15 analyses.

*Loi* loss on ignition.

The pearls were obtained by triplicate in Pt meltpots and collector dishes, using LiI as a viscosity corrector. The spectrometer was calibrated using a set of more than 60 international standards. A separate set of international standards provided by the Geological Survey of Japan was used as an inner control of the quality of results (see for details^66^). Na_2_O was determined by atomic absorption spectroscopy (AAS), with previous total solubilization of the sample (see for detail of analytical procedure^67^). TRS sample was carefully treated at 130 °C in pyrex recipients during 48 h prior to any other manipulation. We consider the valour of LOI of 1 gr of sample obtained in ceramic meltpots running on an oxidizing furnace and considering that the low Fe content of samples and its state of oxidation minimize the possible effect of oxygen uptake during the ignition process^68^).

A CAMECA Camebax SX-50 EMPA-WDS was used for micro-chemical characterizations of zeolites or zeolitic clusters (Table 2). Different natural and synthetic silicates and oxides of certified composition were used as standards (P&H Developments, and Agar Scientific commercial standard blocks). The analysing crystals are whose provided by CAMECA (LIF, TAP and PET) (see for details^69^).

**Table 2:** Microprobe analyses of chabazites and phillipsites (mean values calculated on 8 analyses).

	Chabazites	St. dev	Phillipsites	St. dev
SiO_**2**_	52.53	0.12	54.95	0.14
TiO_**2**_	0.04	0.05	0.01	0.01
Al_**2**_O_**3**_	18.30	0.13	18.90	0.11
Fe_**2**_O_**3**_	0.07	0.02	0.04	0.01
MnO	0.03	0.01	b.d	b.d
MgO	0.08	0.02	b.d	b.d
CaO	5.54	0.8	3.76	0.05
BaO	0.06	0.01	0.02	0.01
Na_**2**_O	0.27	0.01	0.62	0.02
K_**2**_O	6.34	0.8	9.56	0.06
H_**2**_O	17.06	0.9	12.20	0.07
Si	8.52	0.009	11.40	0.007
Ti	b.d	b.d	b.d	b.d
Al	3.50	0.006	4.62	0.005
Fe^**3+**^	0.01	0.002	0.01	0.001
Mn	b.d	b.d	b.d	b.d
Mg	0.02	0.002	b.d	b.d
Ca	0.96	0.007	0.83	0.005
Ba	b.d	b.d	b.d	b.d
Na	0.09	0.002	0.25	0.003
K	1.31	0.003	2.58	0.002
H_**2**_O	9.19	0.090	8.30	0.080
Si/Al	2.93		2.47	
Na/K	0.07		0.10	
CEC theor	3.46		3.61	
CEC calc	1.43		1.54	

Chemical formula is calculated on the base of 32 O for phillipsites and of 24 O for chabazites.

*CEC theor* theorical cation exchange capacity, *CEC calc* calculated cation exchange capacity, *b.d.* below detection limit.

The precision was 3% (1σ) for major elements obtained by XRF and accuracy better than 2%, except Na obtained by ICP-MS. The analytical precision (2σ RSD, n = 4) is 5 to 8% for Na_2_O, and accuracy is better than 4%.

**Figure 1:**
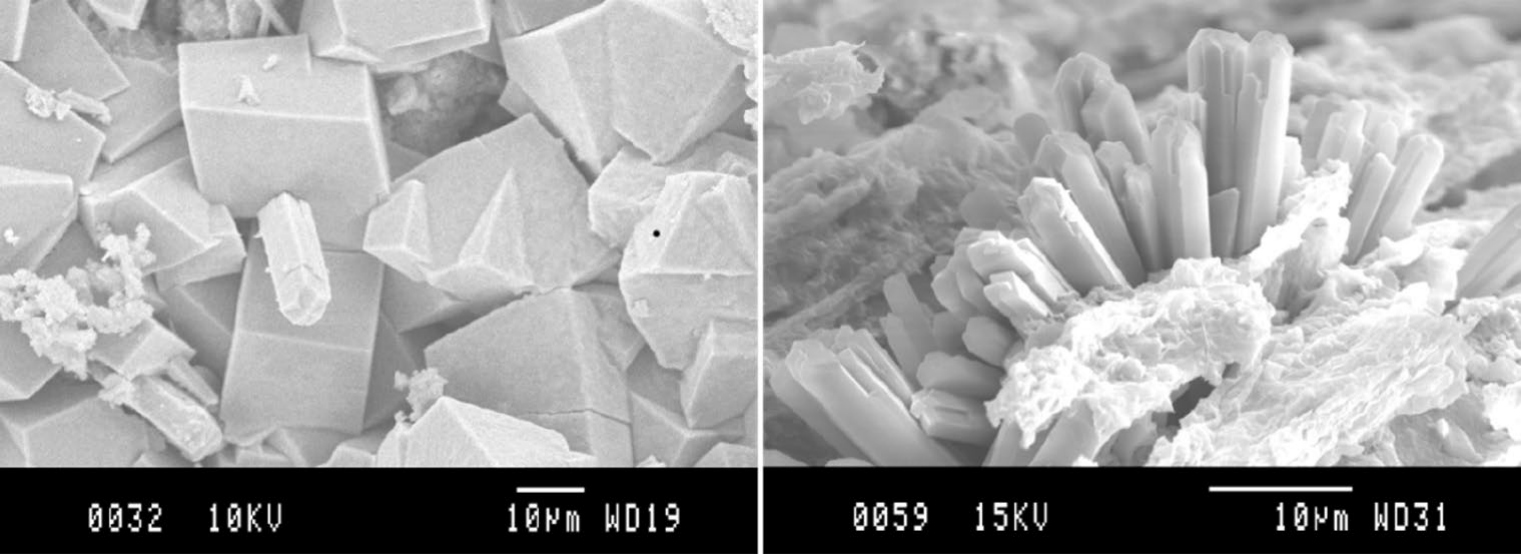
SEM images of the zeolitic phases present in the “Tufo Rosso a Scorie Nere”. Right: phillipsite crystals; Left: chabazite crystals.

Microtextural analysis of zeolites (Fig. 1) were developed on a JEOL J3M-840 scanning electron microscope (SEM). Operating conditions were of 10 kV and a range of variation of 18 to 22 mm in window conditions. Taking into account the special environmental hydration behavior of the samples, previously they were dehydrated in a stove at 60 ºC overnight, then metallized under vacuum by thermal sputtering and preserved again under vacuum prior to their study. We performed a strategy of ultrathin double metallization (first carbon, then gold) to obtain a better quality of image^70^.

Zeolitic water content (Table 2) was calculated by differential thermal analysis (DTA) and thermogravimetry (TG) (Fig. C3, Appendix C) using a Mettler TGA/SDTA851e instrument (10°/min, 30–1100 °C, sample mass of ~ 10 mg, Al_2_O_3_ crucible) according to the method proposed by^71^.

The physical characterization was conducted by the application of gravimetric nitrogen Brunauer–Emmett–Teller (BET) surface analysis technique, using a Micromeritics ASAP 2000 Micropore Analyser. BET analysis provides information about the specific surface area, total pore volume and pore size distribution. Nitrogen adsorption isotherms were obtained at liquid nitrogen temperature (Fig. C4, Appendix C). Prior to the determination of the adsorption isotherm, the sample was outgassed at 300 °C.

### Experimental on San Giustino channel

**Figure 2:**
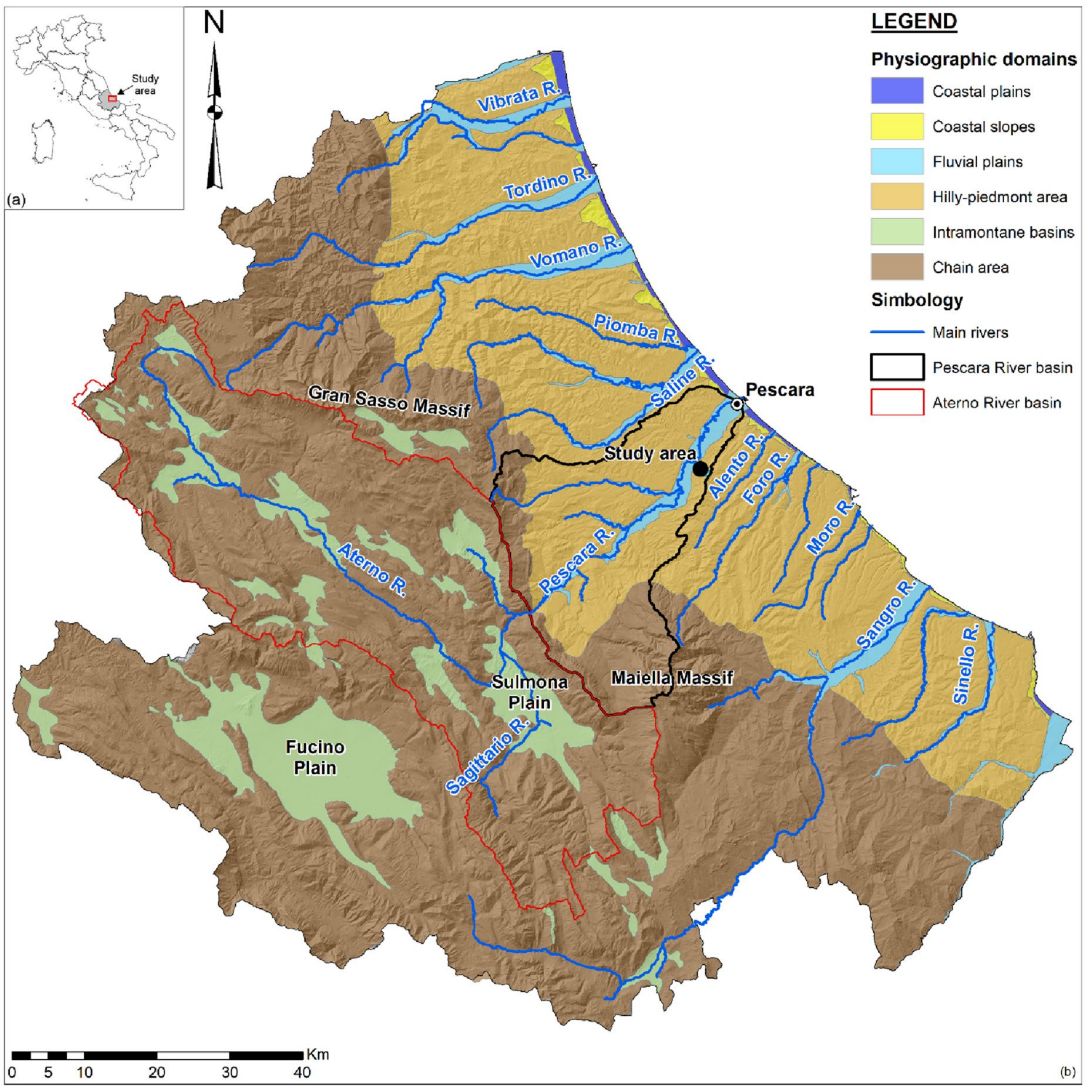
(**a**) Location map of the study area in the Central Italy; (**b**) main physiographic domains of Abruzzo Region. The maps were created by Esri ArcGIS ® 10.6 software (www.esri.com/en-us/store/overview).

A ten-month long campaign developed at San Giustino channel. The San Giustino channel is in the hilly-piedmont area of the Abruzzo Region (Central Italy) and it is a tributary of the Pescara River. Pescara River flows from the eastern slope of Central Apennines (Gran Sasso Massif, 2912 m a.s.l.; Maiella Massif, 2793 m a.s.l.) into port-canal, in the town of Pescara, with a predominantly SW-NE direction. It belongs to the wider Aterno-Pescara River basin and the overall drainage catchment covers a surface area of about 3180 km^2^, of which about 800 km^2^ area in the hilly-piedmont area (Fig. 2).

The area is an important example of a river basin in which artificial/anthropic intervention has profoundly modified the hydrographic network. Among the many landforms created by human activities, there are landfills, sea embankments, motor-way, and railway embankments, airport, open-air quarries or excavation, industrial areas, etc.

From a geological point of view, the Pescara River basin is characterized by the presence of Mio-Plio-Quaternary terrigenous deposits, related to the turbiditic foredeep sequences, unconformably overlain by hemipelagic marine sequences and by Quaternary continental deposits (Fig. B1, Appendix B). The San Giustino channel is mainly constituted by eluvial-colluvial, terraced and alluvial continental deposits and by pelitic-sandy, sandy-pelitic, sandy-conglomeratic marine deposits (Fig. B2, Appendix B). From a mineralogical and geochemical point of view they are constituted by poorly soluble silicates, while the alluvial plain is fed by a limestone-hold aquifers and in shallow sediments directly by rain fall. Evident geogenic sources of high levels of ammonia (i.e. up to 3 mg/L^1^) like humic levels in recent sediments, iron or forests with high rate of vegetal matter recycling are not known in this region.

In anthropized areas without impermeable (clays) sedimentary cover a high degree of vulnerability in superficial unconfined aquifers to the industrial and urban residual waters exists. The main detected pollutant is ammonia related to unauthorized dumping of wastewaters.

During the sampling period the effluent undergone periodical controls and measures of lot of chemical parameters such as concentration of ammonia, nitrates, nitrites, chlorides, calcium, sodium, potassium and magnesium. Also, chemo-physical and physical parameters, such as pH, conductivity and temperature, were periodically monitored (see Table 3). The chemical parameters were measured before and after treatment with zeolitite; in particular, reference is made with the initials "D" to dirty waters (pre-treatment) and with the initials "C" to clean waters (post-treatment).

**Table 3:** Chemical–physical parameters measured at San Giustino channel during the period September–July.

Samplings	NH_4_^+^mg/l	NO_2_^–^mg/l	NO_3_^—^mg/l	Cl^−^ mg/l	Ca mg/l	K mg/l	Na mg/l	Mg mg/l	Cond. S/m	Temp. (°C)	pH
Sept 2 D Sept 2 C	12.99 2.37										
Sept 16 D Sept 16 C	27.90 3.80	0.80 0.01	32 1.80	931 53	25.9 79.0	56 39.5	138 53.4	2.8 20.79			
Sept 23 D Sept 23 C	29 0.16										
Sept 30 D Sept 30 C	29.35 2.33								843	19.4	7.2
Oct 7 D Oct 7 C	40.50 3.89										
Oct 14 D Oct 14 C	30 4.3										
Oct 21 D Oct 21 C	35.40 4.43	0.20 0	18 1.9	49.8 48.1	73.4 90.8	11.9 35.7	56.7 49.1	17.1 19.1			
Oct 28 D Oct 28 C	27.80 1.43										
Nov 4 D Nov 4 C	31.20 1.49										
Nov 11 D Nov 11 C	23.75 2.15								767 747	19.4 19.6	7.03 7.3
Nov 20 D Nov 20 C	74.20 0.05	0.07 0.26	3.1 5.5	51.5 32.5	73.7 76.8	13.7 32.6	58.3 40.6	16.5 16.4	826 631	19.6 19.4	6.9 7.06
Nov 27 D Nov 27 C	23.05 0.20								809 797	19.5 19.6	8 8.47
Dec 2 D Dec 2 C	62.15 3.80								936 809	19.4 19.6	7.53 7.03
Dec 9 D Dec 9 C	19.40 1.35	0.04 0.11	2.4 2.0	34.7 36.5	41.2 51.7	10.7 42.7	24.3 51.4	6.0 9.9	452 576	19.6 19.5	6.96 7.19
Dec 17 D Dec 17 C	66.85 16.68										
Dec 28 D Dec 28 C	40.10 14.93										
Jan 5 D Jan 5 C	31.60 14.89										
Jan 14 D Jan 14 C	62.80 5.14										
Jan 19 D Jan 19 C	4.60 0.02	0.15 0.13	0.01 0.02	11.1 9.1	33.3 28.4	4.96 16.4	14 19.3	5.6 5.2			
Jan 28 D Jan 28 C	7.80 6.49										
Feb 13 D Feb 13 C	115.18 4.52										
Feb 27 D Feb 27 C	99.26 6.62										
Mar 5 D Mar 5 C	111.63 13.83										
Mar 13 D Mar 13 C	18.81 0.11										
Mar 19 D Mar 19 C	110.59 15	0 0	0.01 0.01	59.6 39.6	73.2 73.2	18.6 19.6	18.1 18.2	15.3 15.3			
Mar 26 D Mar 26 C	118.94 6.68										
May 12 D May 12 C	16.88 0.02										
June 7 D June 7 C	11.55 10.6										
July 11 D July 11 C	11.18 4.22										

*D* dirty waters, *C* clean waters.

### Column continuous experiment

Preliminary treatments of the zeolitic rock regarded grounding and sieving so as to obtain dimensions ranging from 0.3 to 0.6 mm; regarding this point, it can be stated that particle size has a strong effect on the breakthrough capacity of the zeolitite; some study^72^ found that 0.5–1 mm particle size gave the highest performance, while others^26,73^ stated that small particle size increased the removal efficiency due to the fact that, as the particle size decreases, the surface area and sorption capacity increase. In all semi-industrial and industrial plants, the focus is in finding a balance between the ideal grain size for ammonia uptake and the practical procedure concerns (i.e., cleaning and regeneration of zeolitite versus periodic obturation of pipes by fine-grained sediments).

The NH_3_ removal experiments were conducted using a flow system with a glass column (Pyrex glass, 10 mm internal diameter) at ambient temperature. The column was filled with the zeolitite powder (100 g), and the following reagents were added: 100 ml of 600 mg/l NH_4_Cl, 20 ml NaOH 20%, 50 ml H_2_SO_4_ (0.05 N); the starting effective NH^4+^ concentration, was of 211.07 mg/l. Water flow rate, controlled with a pump, was fixed to 0.4 l/s to simulate the mean value measured at San Giustino channel. Table 4 summarizes the physical characteristics of the experimental system.

**Table 4:** Characteristics of the column experimental system.

Characteristics of the experimental system	
Water flow rate	0.4 l/s
Column internal diameter	35.5 mm
Packet height	110 mm
Bed volume	109 cm^3^
Mass of zeolitite	100 gr

**Figure 3:**
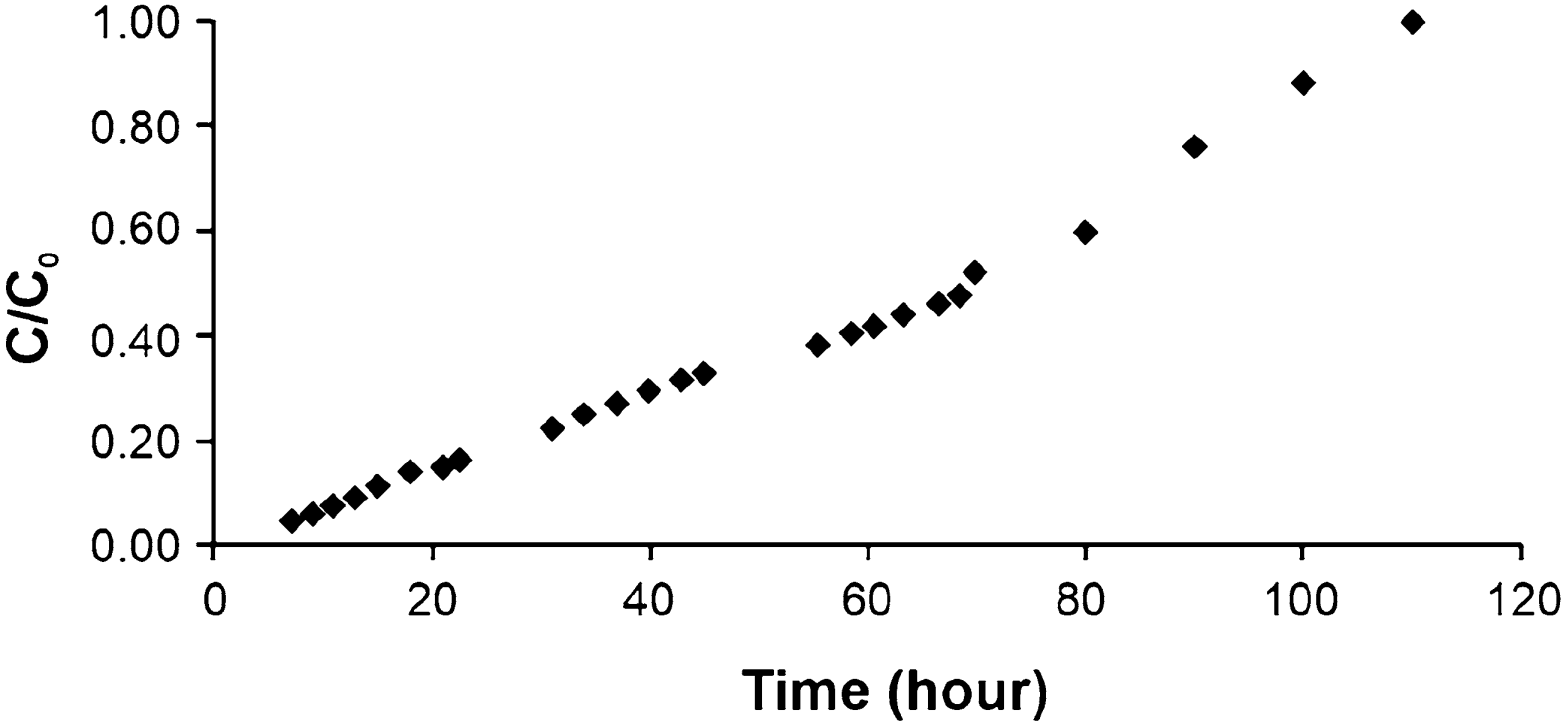
Breakthrough curve for water containing NH^4+^ ions. C_0_ = initial NH^4+^ concentration.

**Figure 4:**
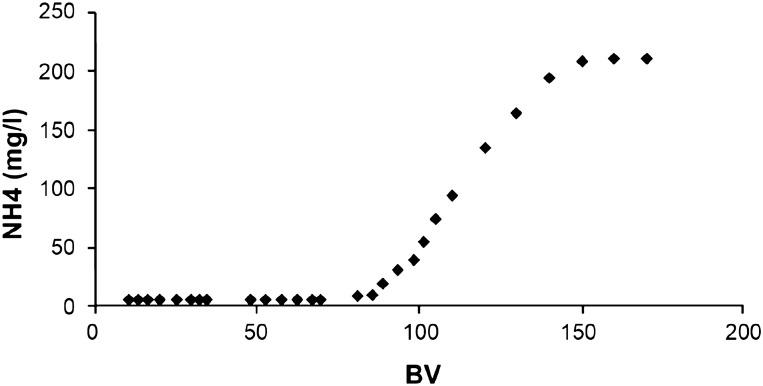
NH^4+^ (mg/l) vs number of liters of water passing through the experimental column. BV = ratio between the volume of solution and the volume of zeolitite.

The column loading capacity, i.e., the zeolitite saturation, was determined from the breakthrough curve (Fig. 3). Figure 4 illustrates the efficacy of the zeolitite in the removal of NH^4+^ expressed as number of liters of water passing through the experimental column.

### Pilot plant on San Giustino channel

**Figure 5:**
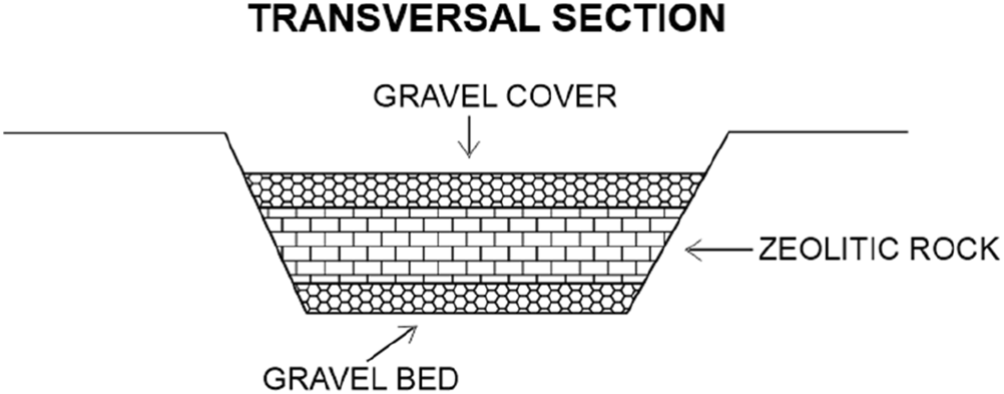
Schematic transversal section of the pilot plant built up at San Giustino channel.

The ion exchange tests were carried out in a small stretch of about 70 m of the San Giustino canal. The tests were performed using 25 m^3^ of zeolitite, placed on the bed of the watercourse characterized by an average flow rate of about 0.4 l/s. Figure 5 illustrates the scheme of the pilot plant built up at the San Giustino channel. A zeolitite bed 20 cm in height was positioned on a portion of 70 m in length and 70 cm in width of the effluent; this short course was characterised by a mean water flow rate of 0.4 l/s. The total amount of the zeolitite used for the pilot plant was of 25 m^3^. Seven zeolitic beds were positioned along the effluent and intercalated by decantation and homogenization baths.

## Results and discussion

XRD analyses conducted on an average of 15 TRS samples revealed small variations in the percentage of minerals that constitute the zeolitite. However, the data to which attention must be paid is the zeolitic content of the zeolitite, that is the sum of chabazite and phillipsite. In fact, only the zeolite fraction is responsible for the cation exchange performance for ammonia removal. Quantitative analyses conducted on an average of 15 samples revealed an average degree of zeolitization of 60%. Figures C1 and C2 and Table C1 of Appendix C show the results of the XRD analysis and related QPA analysis of an exemplary sample of TRS powder. Results of PXRD analysis on TRS reveal a mineralogical composition made of chabazite, phillipsite, sanidine and volcanic glass (Fig. C1, Appendix C). Quantitative weight percentages of minerals resulted in 52.15% ± 2% chabazite, 8.45% phillipsite ± 1%, 20.62% ± 2% sanidine and 18.78% ± 2% volcanic glass (Table C1, Appendix C). A final whole zeolitization degree of 60.60% is reported.

Chabazite appears as pseudocubic crystals (of about 25 µm) and frequently in aggregates in TRS matrix (Fig. 1); phillipsite appears as acicular clusters (diameter of about 40 µm) constituted by thin prismatic crystals growing inside scoriae. The BET surface area results of 19.5 m^2^ g^−1^. The nitrogen adsorption isotherms of the studied samples (Fig. C4, Appendix C) are of Type IV according to IUPAC classification which is typical for mesoporous materials. The observed pore sizes correspond to mesopores^74^. Average pore size is 14,3 nm, and the volume pore size is 0,06 cm^3^ g^−1^. All these results are in good agreement with the ones obtained for zeolitite of the same sampling site (^75^ Table 1, Z1 sample).

Zeolitic water content, calculated by DTA and TG analysis (Fig. C3, Appendix C) is reported in Table 1.

The theoretical ionic exchange capacity (CEC) of chabazite and phillipsite are around 3.5 mequiv/g (see Table 2); the real capacity, anyway, is significantly lower (1.5 mequiv/g), being it affected by the concentration of the ion to be removed compared with the ionic strength.

As above stated, column experiments were performed to calculate the saturation time of the zeolitite from the breakthrough curve.

By analysing the results of the column experiments, it results that the time required to reach saturation of the zeolitite is of about 110 h (Fig. 3). The ability of zeolitite to adsorb ammonia is, infact, not unlimited and once it reaches saturation, it can be placed into a salt water solution to be recharged. This charging and removing of ammonia from zeolite can be repeated many times prior to the zeolite become clogged and useless^76^. Moreover, the efficacy of the zeolitite bed is guaranteed till the passage of about 80 l of water as is visible from Fig. 4. For these results and taking also into account the geometry of the San Giustino channel, a total amount of 25 m^3^ of zeolitite was estimated as necessary to guarantee water purification process over a period of ten months.

**Figure 6:**
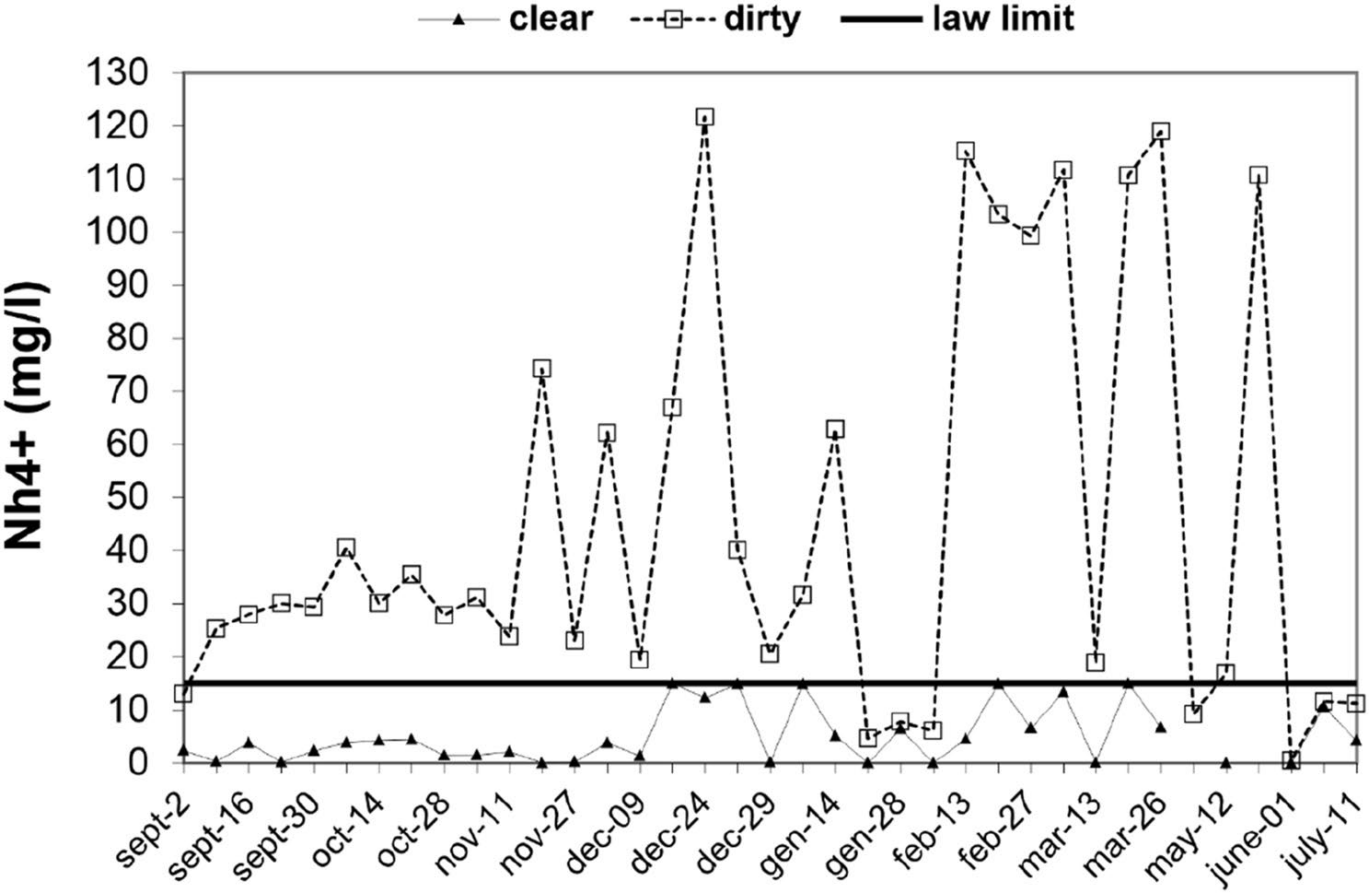
Ammonia removal expressed as variation in NH^4+^ (mg/l) during the period September–July.

By analysing the results of the ten long period monitoring of the chemical-physical parameters at San Giustino channel, it results that NH^4+^ input values range between 5 and 120 mg/l (Table 3). Figure 6 shows the results of the ammonia removal on the pilot plant at San Giustino channel during the period between September and July.

As it is clearly visible, the efficacy of the zeolititic bed is proved, being the NH^4+^ values always under the law limit of 15 mg/l. Results for clear waters indicate NH^4+^ values under 10 mg/l, very often ranging between 1 and 5 mg/l. At the end of the experimentation, it can be stated that the calculated 25 m^3^ amount of zeolitite used for the process resulted more than sufficient to guarantee water purification; in fact, it also bore occasional surplus in wastewaters which caused increasing in flow rate as sometimes happened during the ten months long period.

Also, the increasing in rainfall sometimes caused a rising in the flow rate which reached values of about 30/40 l/s; this excess was regulated through the insertion of by-pass systems characterised by a 2.5 l/s flow rate.

## Conclusions

In this study NH^4+^-N removal from a little wastewater course (San Giustino channel, Abruzzo Region, Central Italy) by using a local chabazite-phillipsite zeolitite was investigated. The course is characterised by NH^4+^ input values ranging between 5 and 120 mg/l. A laboratory preliminary study finalised to determine the efficacy of the zeolitite in the ammonia removal was conducted in a column system test. The induced flow rate was of 0.4 l/s, being this the mean flow rate value measured at San Giustino channel over a ten-long period. By analysing the results of the breakthrough curve, it results that the system saturates in a long period, about 110 h, which correspond to the passage of about 80 l of water. Taking into account these findings and the geometry of the San Giustino channel water course, a total amount of 25 m^3^ of zeolitite was calculated to be necessary to guarantee ammonia removal under the limit laws over a ten-month long period. This prevision was confirmed by the results of the ammonia removal campaign, NH^4+^ concentration values always resting under the law limit. This experience might be considered just an example of the simplicity and economy of the use of this variety of zeolite in the removal of ammonia uptake and removal from aqueous effluents.

In summary, industrial use of natural zeolites (zeolitites) versus synthetic ones usually is limited by its natural variability, instead of the good monophase (or relatively stable biphase percentages) of synthesis products. The commonly used term “natural zeolite” corresponds to a rock mainly constituted by several zeolite (and in a lesser degree other) mineral phases and eventually residual glass, and the term “zeolitite” for this rock should be imposed to avoid misinterpretations.

We propose henceforth the use of a classification system for the absorbent properties of zeolite, regardless of its mineralogical composition. This could be done by considering the CEC of pure phillipsite and considering expressing the CEC of any studied zeolitite in relative percentual of equivalent (pure) phillipsite. The use of this procedure would serve to standardize the characteristics of a zeolite both in a scientific article and in the trade of this product.

However chemical composition and CEC is not enough to characterize a zeolitite and each supplier should provide the quantitative mineral composition obtained by PXRD of the traded rock; only this data allows for comparison between zeolitite from several deposits and suppliers. Only quantitative PXRD characterization allows for predictive evaluation of the potential use of a zeolitite. Terms like “Italian zeolite”, “Japanese zeolite”, “Turkish zeolite”, etc., that have proliferated in the technical literature are incorrect, not descriptive, and misleading, since important mineral variation exists even at the scale of a single minable deposit (in mineral composition, and in percentage composition of each mineral phase). Zeolitite suppliers should provide the quantitative (a range of, as narrow as possible) mineral composition of its products, and editors from scientific and technical journals should take care that these data are clearly specified in each published research, since without this information the researches are not directly comparable and reproducible for other people. In addition, absence of these data hurts several specific industrial uses of zeolitite, since reiterate artisanal adjustments and controls in operational plants are required. In the same way that nobody would accept uncertified and variable compositions of synthetized zeolites for advanced technical purposes, zeolitite rock expansion in industry requires more standardized and precise mineral composition data.

The review of published studies shows that ammonia uptake from water is overwhelmingly focused on clinoptilolite (and minor mordenite)-rich zeolitites, in despite of well-known suitability (and frequently, better results, up to 61–79% improvement of ammonia uptake) of phillipsite-chabazite rich rocks. This how-know seems essentially to have been over decades an “Italian matter”. The purpose of this research has been to remind to a larger public that phillipsite-chabazite is a widespread zeolitite resource, and that good business opportunities exist for geological prospecting and mining of phillipsite-chabazite rich zeolitites in many geological regions and countries in continents and oceanic islands. Therefore, local supply for cheap and effective zeolitite rocks for a large variety of industrial applications might be envisaged in these countries, starting from cleaning and reuse of wastewater, marine water desalination, etc. Future research in this field related to phillipsite-chabazite zeolitite might be focused in the evaluation of the regeneration processes of this raw material, total effective cycles of use, and ratio of performance of these cycles. Last but not least parallel research during this prosecution of the study might be focused to the circular-economy production of synthetic N-rich fertilizers during regeneration of phillipsite-chabazite zeolitite.

## Supplementary Information

Supplementary Information.

## Data Availability

All data generated or analysed during this study are included in this published article [and its supplementary information files].
