# Supplementary material for: On the suitability of phillipsite-chabazite zeolitite rock for ammonia uptake in water: a case study from the Pescara River (Italy)
Source: Sci Rep. 2022 Jun 3;12:9284. doi: 10.1038/s41598-022-13367-y (PMC9166697; doi:10.1038/s41598-022-13367-y)
Supplement: Supplementary file 1 — Supplementary Information. [file 41598_2022_13367_MOESM1_ESM.docx]

**Appendix A: (non-exhaustive) list of works related to the subject of the paper mainly based on the use of clinoptilolite (not referred in the main draft)**

Almutairi, A.; Weatherley, L. R. Intensification of ammonia removal from waste water in biologically active zeolitic ion exchange columns. *Journal of Environmental Management* **2015,** 160: 128-138.

Alshameri, A.; Yan, C.; Al-Ani, Y.; Dawood, A. S.; Ibrahim, A.; Zhou, C.; Wang, H. (2014) An investigation into the adsorption removal of ammonium by salt activated Chinese (Hulaodu) natural zeolite: Kinetics, isotherms, and thermodynamics. *Journal of the Taiwan Institute of Chemical Engineers* **2014**, 45: 554–564.

Beebe, D. A.; Castle, J. W.; Rodgers Jr. J. H. Treatment of ammonia in pilot-scale constructed wetland systems with clinoptilolite. *Journal of Environmental Chemical Engineering* **2013,** 1: 1159–1165.

Cooney, E. L.; Booker, N. C.; Shallcross, D. C.; Stevens, G. W. Ammonia Removal from Wastewaters Using Natural Australian Zeolite. II. Pilot-Scale Study Using Continuous Packed Column Process. *Separation Science and Technology* **1999**, 34:14, 2741-2760, DOI: 10.1081/SS-100100802

Cruz, H.; Law, Y. Y.; Guest, J. S.; Rabaey, K.; Batstone, D.; Laycock, B.; Verstraete, W.; Pikaar, I. Mainstream Ammonium Recovery to Advance Sustainable Urban Wastewater Management. *Environ. Sci. Technol.* **2019**, 53, 11066−11079.

Vaičiukynienė, D.; Mikelionienė, A.; Baltušnikas, A.; Kantautas, A.; Radzevičius, A. Removal of ammonium ion from aqueous solutions by using unmodified and H2O2-modified zeolitic waste. *Scientific Report*s **2020,** 10:352, <https://doi.org/10.1038/s41598-019-55906-0>

Englert, A. H.; dos Santos, C. C.; Gobbi, S. A.; Rubio, J. Adsorbing flocs in expanded/fluidised bed reactors: A new basis for pollutants removal. *Minerals Engineering* **2006,** 19: 918–924.

Erdoğan, B. C.; Ülkü, S. Ammonium sorption by Gördes clinoptilolite rich mineral specimen. *Applied Clay Science* **2011,** 54: 217–225.

Zsófia Ganrot Chapter 17: Use of Zeolites for Improved Nutrient Recovery from Decentralized Domestic Wastewater. In *Handbook of Natural Zeolites* ; Inglezakis, V. J., Zorpas, A. A. Eds.; Bentham Science Publishers, Sharjah UAE 2012; pp 410-435.

Guaya, D.; Valderrama, C.; Farran, A.; Armijos, Ch.; Cortina, J. L. Simultaneous phosphate and ammonium removal from aqueous solution by a hydrated aluminum oxide modified natural zeolite. *Chemical Engineering Journal* **2015,** 271: 204–213.

Guida, S.; Potter, C.; Jefferson, B.; Soares, A. Preparation and evaluation of zeolites for ammonium removal from municipal wastewater through ion exchange process. *Scientific Reports* **2020,** 10: 12426 https://doi.org/10.1038/s41598-020-69348-6

Hrenovi, J.; Büyükgüngör, H.; Orhan, Y. Use of Natural Zeolite to Upgrade Activated Sludge Process. *Food Technol. Biotechnol.* **2003,** 41 (2): 157–165.

Kotoulas, A.; Agathou, D.; Triantaphyllidou, I. E.; Tatoulis, T. I.; Akratos, C. S.; Tekerlekopoulou, A. G.; Vayenas D. V. Zeolite as a Potential Medium for Ammonium Recovery and Second Cheese Whey Treatment. *Water* **2019,** 11, 136, 23 pp. doi:10.3390/w11010136.

Liberti, L.; Limoni, N.; Lopez, A.; Passino, R.: Boari, G. The 10 m^3^ h^−1^ rim-nut demonstration plant at West Bari for removing and recovering N and P from wastewater *Water Research* **1986,** 20 (6):735-739.

Malovanyy, A.; Sakalova, H.; Yatchyshyn, Y.; Plaza, E.; Malovanyy, M. Concentration of ammonium from municipal wastewater using ion exchange process. *Desalination* **2013,** 329 93–102.

Mercer, B. W.; Ames, L. L. Zeolite ion exchange in radioactive and municipal wastewater treatment. In *Natural zeolites. Occurrence, properties, use*; Sand L. B., Mumpton F. A. Eds.; Pergamon Press Oxford 1978; pp 451-462.

Murphy, C. B.; Hrycyk, O.; Gleason, W. T. Natural zeolites: novel uses and regeneration in wastewater treatment. In *Natural zeolites. Occurrence, properties, use*; Sand L. B. , Mumpton F. A. Eds.; Pergamon Press Oxford 1978; pp 471-478.

Nguyen M. L.; Tanner C. C. Ammonium removal from wastewaters using natural New Zealand zeolites, *New Zealand Journal of Agricultural Research* **1998**, 41:3, 427-446.

Park, J. B.; Lee, S. H.; Lee, J. W.; Lee, C. Y. Lab scale experiments for permeable reactive barriers against contaminated groundwater with ammonium and heavy metals using clinoptilolite (01-29B). *Journal of Hazardous Materials* **2002,** B95: 65–79.

Sancho, I.; Licon, E.; Valderrama, C.; de Arespacochaga, N.; López-Palau, S.; Cortina, J. L. Recovery of ammonia from domestic wastewater effluents as líquid fertilizers by integration of natural zeolites and hollow fibre membrane contactors. *Science of the Total Environment* **2017,** 584–585: 244–251.

Semmens M. J.; Porter, P. S. Ammonium Removal by Ion Exchange: Using Biologically Restored Regenerant. *Water Pollution Control Federation* **1979,** 51 (12): 2928-2940.

Shaban,M.; AbuKhadra, M. R.; Nasief F. M.; Abd El-Salam, H. M. Removal of Ammonia from Aqueous Solutions, Ground Water, and Wastewater Using Mechanically Activated Clinoptilolite and Synthetic Zeolite-A: Kinetic and Equilibrium Studies. *Water Air Soil Pollut* **2017,** 228: 450. https://doi.org/10.1007/s11270-017-3643-7

Wang, Y.F.; Lina, F.; Pang, W.Q. Ammonium exchange in aqueous solution using Chinese natural clinoptilolite and modified zeolite. *Journal of Hazardous Materials* **2007,** 142: 160–164.

Wang, Y.; Kmiya, Y.; Okuhara, T. Removal of low-concentration ammonia in water by ion-exchange using Na-mordenite. *Water Research* **2007,** 41: 269-276.

Weatherley, L. R.; Miladinovic, N. D. Comparison of the ion exchange uptake of ammonium ion onto NewZealand clinoptilolite and mordenite. *Water Research* **2004**, 38: 4305-4312.

Widiastuti, N.; Wu, H.; Ang, H. M.; Zhang, D. Removal of ammonium from greywater using natural zeolite. *Desalination* **2011,** 277: 15–23.

**Appendix B**: **Geological setting of the study area supported by geological maps and related references.**

**Geological setting of the study area**

The San Giustino channel is in the hilly-piedmont area of the Abruzzo Region (Central Italy) and it is a tributary of the Pescara River (see main text Fig. 2 therein).

The hilly-piedmont area is characterized by a low-relief hilly landscape carved on sin- and late-orogenic Mio-Plio-Quaternary terrigenous deposits, related to the turbiditic foredeep sequences of the relative marine basin, largely covered and unconformably overlain by hemipelagic marine sequences (i.e., clay, sand, and conglomerate), and by Quaternary continental deposits (mainly colluvial, fluvial, and alluvial deposits) (Fig. B1). The present-day geomorphological setting is resulted from the combination of regional uplifting and eustatic sea-level fluctuations, together with geomorphological and environmental processes, climate changes in the past, and the related variation in sediment transport [1-3]. These processes have controlled the selective erosion on hills and slopes and the shaping of river valleys, with the formation of a series of wide alluvial fans and fluvial terraces. This strongly contributed to define the arrangement of the drainage network and induced strong selective erosion processes.

The Pescara River basin reaches its maximum altitude along the eastern front of the chain area and then, the morphology gradually drops down from the piedmont area to-wards the coastal sector. The lower part of the basin shows a main alluvial valley, limited by steep slopes on the right side and large gentle slopes on the left one, and it is interrupted by urban areas and coastal hydrographical arrangement. The main alluvial valley is characterized by fluvial and alluvial deposits arranged in five main orders of entrenched terraces, commonly named T1 to T5, starting from the highest. The drainage network generally shows a sinuous to meandering drainage pattern. It is characterized, at the local scale, by subparallel drainage patterns (mainly in the lower sectors), by trellis patterns (predominant in both the western and eastern sectors), and by an angular pattern, on the central sector. Local convergent, radial, and sub-dendritic patterns are related to isolated reliefs [4,5] (Fig. B1).


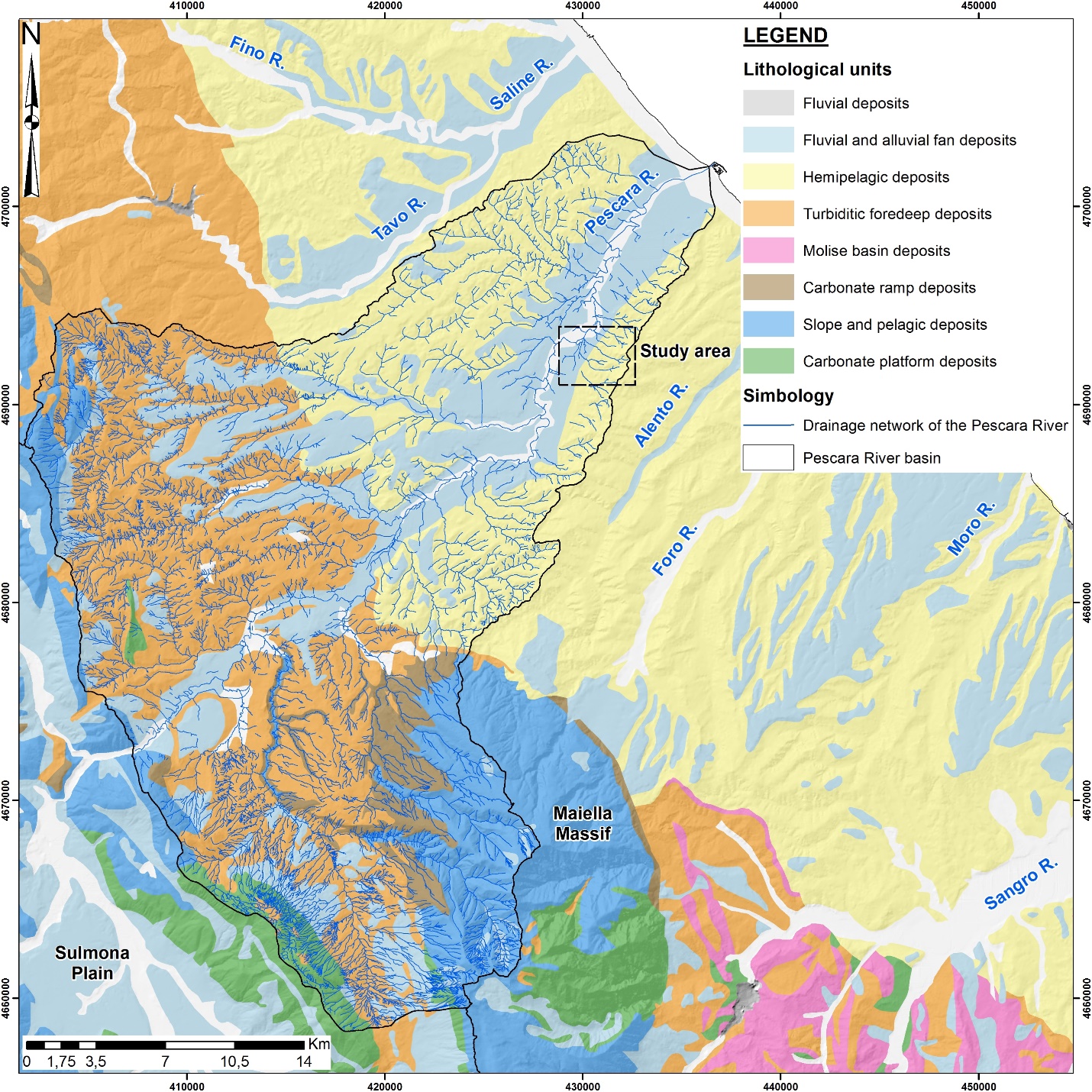


**Figure B1**. Lithological sketch map of Central-eastern Abruzzo Region. The sketch of lithological map was created by Esri ArcGIS ® 10.6 (www.esri.com/en-us/store/overview).

The San Giustino channel flows in SE-NW direction, towards the Pescara River, and it characterized by eluvial-colluvial, terraced and alluvial deposits (Fig. B2). The eluvial-colluvial deposits are formed by very loose to moderately consolidated deposits made up of silt, clay, sand, and reworked soil sediments with thickness ranging from a few to 10 m. The terraced deposits are distinguished in mainly sandy deposits and mainly gravelly deposits. They are made up of sands, silts, and interbedded gravels, arranged in levels and lenses. Finally, the alluvial plain is formed by loose gravel with small levels and lenses of coarse to medium sand with thickness <3m. The bedrock includes three main units of marine deposits: pelitic-sandy, sandy-pelitic, and sandy-conglomeratic deposits referable to Mutignano Fm. [6, 7,8].

In the hilly-piedmont sector of the Pescara River basin, the aquifer is contained into the terraced deposits and the current alluvial plain. It is fed by the limestone aquifers of the Apennine chain in the internal areas, while recharge from rainfall has a major role in the total recharge to the shallow alluvial aquifers moving downstream towards the sea, particularly in correspondence of the terraced deposits that are hydraulically connected to the lower valleys [9]. The permeability of alluvial deposits is variable from 10-3 to 10-4 m/s. The salinization of groundwaters is connected to the presence of depressions of the piezometric surface, in its turn connected to the development for industrial and irriguous purpose, while the extension of the saltwater wedge depends mostly by the permeability and the groundwaters discharge [10]. Finally, the anthropic system between Popoli Gorges and the Adriatic coast may constitute real and potential pollution causes for the groundwater. In fact, near the coastal plain and the main industrial areas, the worst groundwaters are present. A high degree of vulnerability is defined where the thickness of the silty-clayey cover is reduced or absent. In these areas, the main industrial centers that contribute to the increase of the risk of pollution of the aquifer are located [10]. Pollution at the San Giustino channel course is mainly due to unauthorised dumping of wastewaters by the neighbouring built-up areas which spill pollutants in this course even if a purification plant is present at a very short distance. The main pollution is represented by the accumulation over the law limit of ammonia.


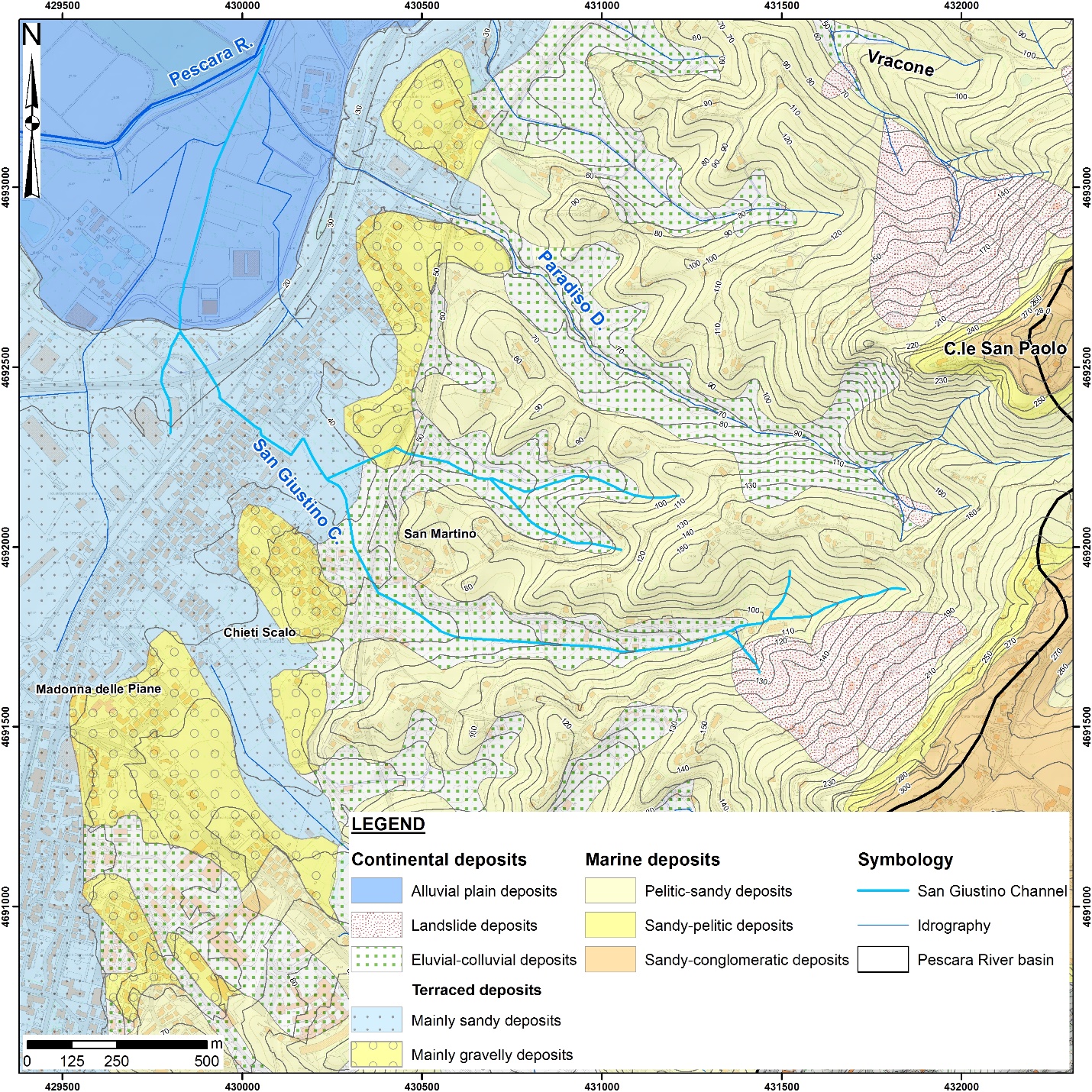


**Figure B2.** Geological map of San Giustino channel. The geological map was created by Esri ArcGIS ® 10.6 software (www.esri.com/en-us/store/overview).

**References**

1. D'Alessandro, L.; Miccadei, E.; Piacentini, T. Morphostructural elements of central–eastern Abruzzi: contributions to the study of the role of tectonics on the morphogenesis of the Apennine chain. In: "Uplift and erosion: driving processes and resulting landforms", International workshop, Siena, September 20-21, 2001. Quat. Int. 2003, 101-102, 115-124, Elsevier Science Ltd and INQUA, Oxford U.K., doi:10.1016/S1040-6182(02)00094-0.
2. Parlagreco, L.; Mascioli, F.; Miccadei, E.; Antonioli, F.; Gianolla, D.; Devoti, S.; Leoni G. Holocene Relative Sea Level Rise along the Abruzzo coast (western central Adriatic). Quat. Int. 2011, 232, 179-186, doi: 10.1016/j.quaint.2010.07.021.
3. Pasculli, A.; Palermi, S.; Sarra, A.; Piacentini, T.; Miccadei, E. A modelling methodology for the analysis of radon potential based on environmental geology and geographically weighted regression. Environmental Modelling & Software 2014, 54, 165-181, ISSN 1364-8152, http://dx.doi.org/10.1016/j.envsoft.2014.01.006.
4. Crescenti, U. Note illustrative della Carta Geologica d’Italia alla scala 1:50,000, Foglio 361 ‘Chieti’. Servizio Geologico d’Italia, ISPRA 2012, 1-102.
5. Ori, G.G.; Rusciadelli, G. Note illustrative della Carta Geologica d’Italia alla scala 1:50,000, Foglio 351 ‘Pescara’. Servizio Geologico d’Italia, ISPRA 2012, 1-118.
6. Parotto, M.; Cavinato, G.P.; Miccadei, E.; Tozzi, M. Line CROP 11: Central Apennines. CROP Atlas: Seismic reflection profiles of the Italian crust. Mem. Descr. della Cart. Geol. d’Ital. 2003, LXII, 145–163.
7. ISPRA (2010a). Carta Geologica d’Italia alla scala 1:50,000, Foglio 351 ‘Pescara’. Servizio Geologico d’Italia. Retrieved from http://www.isprambiente.gov.it/%0AMedia/carg/351_PESCARA/Foglio.html
8. ISPRA (2010b). Carta Geologica d’Italia alla scala 1:50,000, Foglio 361 ‘Chieti’. Servizio Geologico d’Italia. Retrieved from http://www.isprambiente.gov.it/%0AMedia/carg/361_CHIETI/Foglio.html
9. Chiaudani, A.; Di Curzio, D.; Palmucci, W.; Pasculli, A.; Polemio, M.; Rusi, S. Statistical and Fractal Approaches on Long Time-Series to Surface-Water/Groundwater Relationship Assessment: A Central Italy Alluvial Plain Case Study. Water 2017, 9, 850, doi:10.3390/w9110850.
10. Desiderio, G.; Nanni, T.; Rusi, S. La pianura alluvionale del fiume Pescara (Abruzzo): idrogeologia e vulnerabilità dell’acquifero. Mem. Soc. Geol. It. 2001, 56, 197-211.

**Appendix C: experimental procedures.**


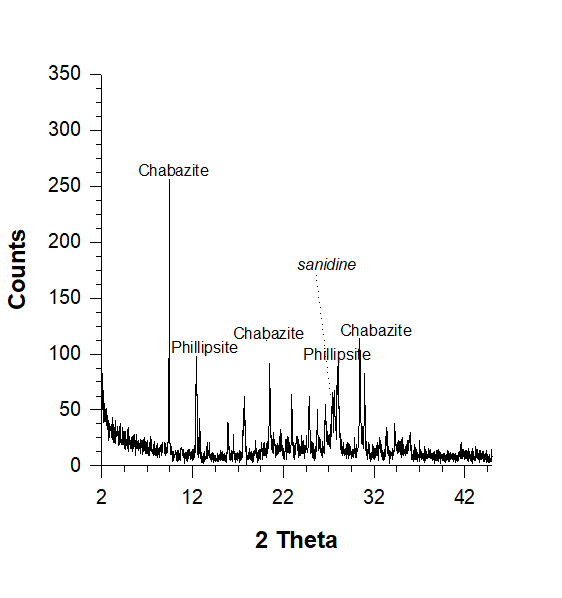


**Figure C1**: XRD spectrum of the zeolitite.


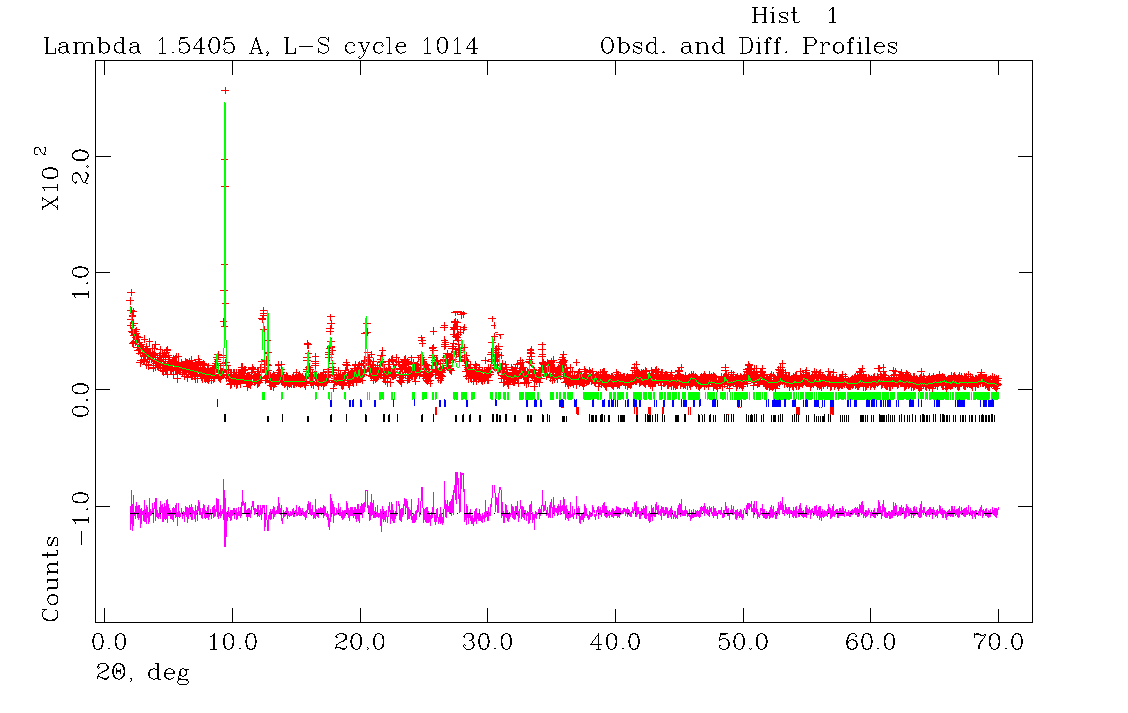


**Figure C2:** Rietveld refinement plots: Observed (red +) and calculated (green) profiles and difference plot (pink) for zeolitite and corundum NIST 676a with tick marks at the positions of the Bragg peaks. From the bottom: chabazite, corundum NIST 676a, sanidine and phillipsite.


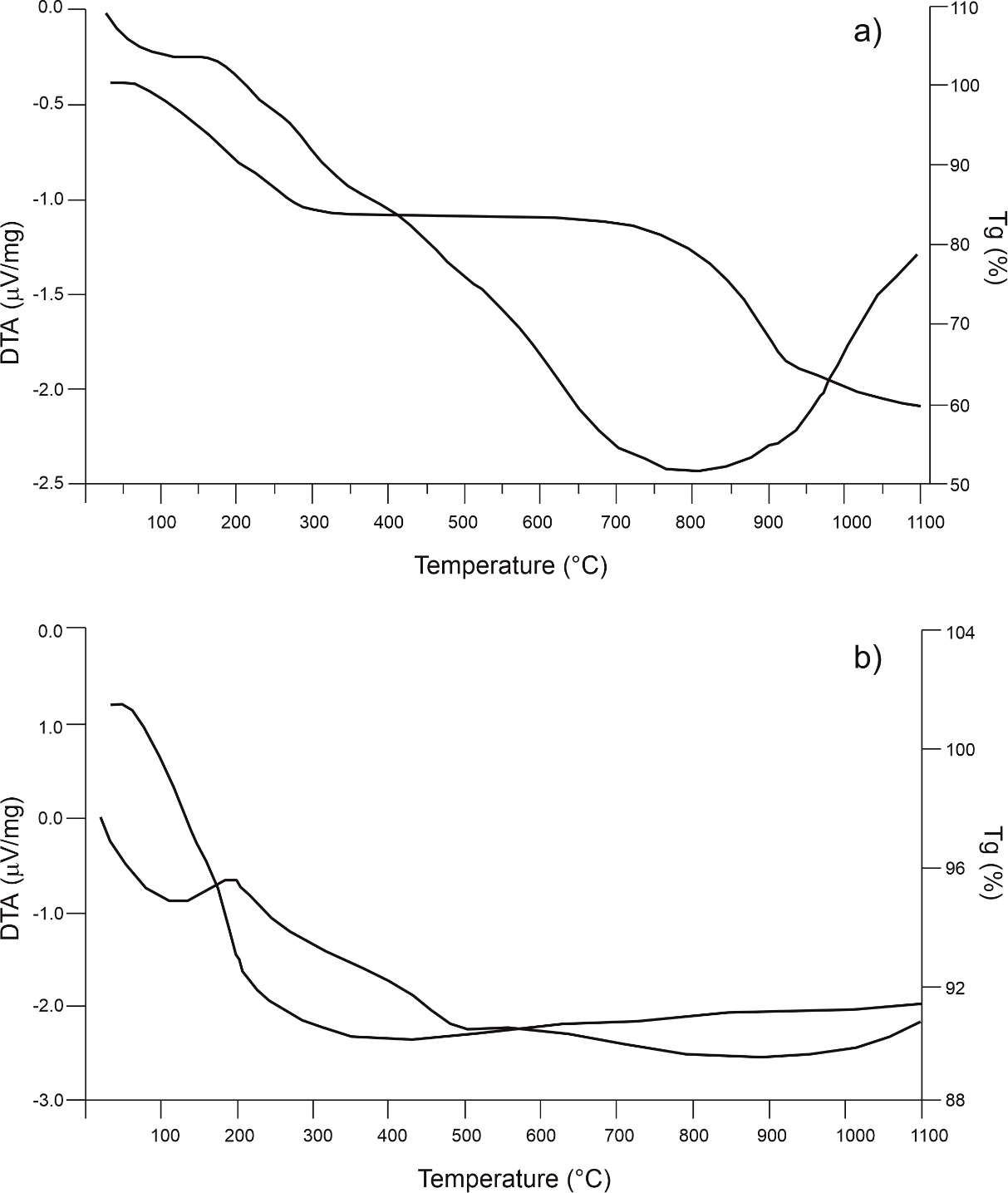


**Figure C3:** Differential thermal analysis and Thermogravimetry for chabazite (a) and phillipsite (b) crystals.


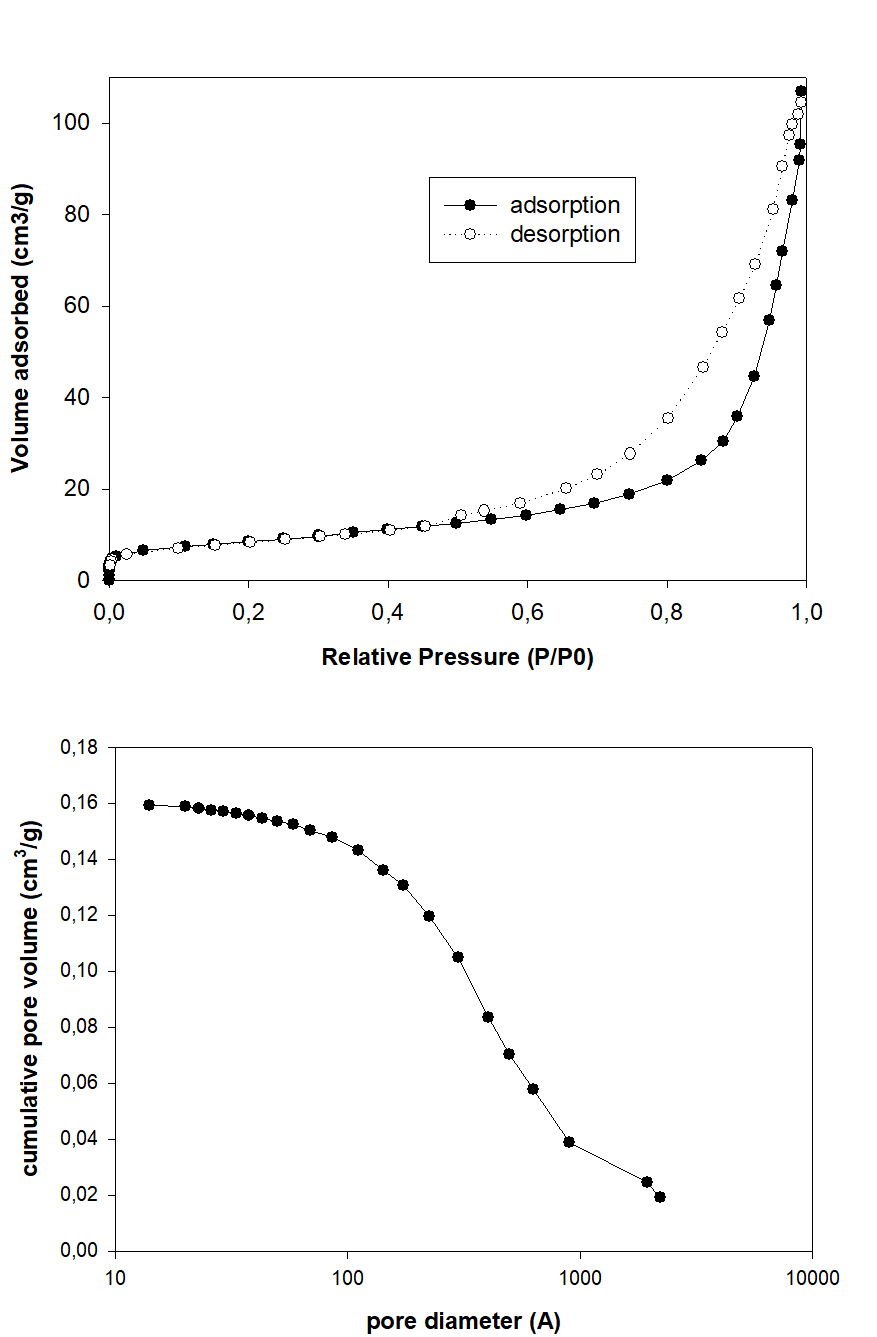


**Figure C4:** Nitrogen adsorption-desorption isotherm (above) and corresponding pore diameter distribution pattern (belowe).

**Table C1:** Rietveld fit results.

|  | **zeolitite** |
| --- | --- |
| **Wavelenght (Å)** | 1.5418 |
| **No. of observation** | 1014 |
| ***R_wp_*** | 0.18 |
| ***R_p_*** | 0.12 |
| ***CHI^2^*** | 1.51 |
| **% amorphous** | 18.78(± 2%) |
| **% phase chabazite** | 52.15(± 2%) |
| **% phase phillipsite** | 8.45(± 1%) |
| **% phase sanidine** | 20.62(± 2%) |
